# *In silico* analysis of the polygalacturonase inhibiting protein 1 from apple, *Malus domestica*

**DOI:** 10.1186/s13104-015-1025-z

**Published:** 2015-03-11

**Authors:** Lerato BT Matsaunyane, Dean Oelofse, Ian A Dubery

**Affiliations:** Agricultural Research Council - Vegetable and Ornamental Plant Institute (ARC-VOPI), Roodeplaat, Pretoria, South Africa; Department of Biochemistry, University of Johannesburg, P.O. Box 524, Auckland Park, 2006 South Africa

**Keywords:** Disease resistance, PGIP, Polygalacturonase, Inhibitor, Structure

## Abstract

**Background:**

The *Malus domestica polygalacturonase inhibiting protein 1* (*MdPGIP1*) gene, encoding the *M. domestica* polygalacturonase inhibiting protein 1 (*Md*PGIP1), was isolated from the Granny Smith apple cultivar (GenBank accession no. DQ185063). The gene was used to transform tobacco and potato for enhanced resistance against fungal diseases.

**Findings:**

Analysis of the *MdPGIP1* nucleotide sequence revealed that the gene comprises 993 nucleotides that encode a 330 amino acid polypeptide. *In silico* characterization of the *Md*PGIP1 polypeptide revealed domains typical of PGIP proteins, which include a 24 amino acid putative signal peptide, a potential cleavage site [Alanine-Leucine-Serine (ALS)] for the signal peptide, a 238 amino acid leucine-rich repeat (LRR) domain, a 46 amino acid N-terminal domain and a 22 amino acid C-terminal domain. The hydropathic evaluation of *Md*PGIP1 indicated a repetitive hydrophobic motif in the LRR domain and a hydrophilic surface area consistent with a globular protein. The typical consensus glycosylation sequence of Asn-X-Ser/Thr was identified in MdPGIP1, indicating potential N-linked glycosylation of *Md*PGIP1. The molecular mass of non-glycosylated *Md*PGIP1 was calculated as 36.615 kDa and the theoretical isoelectric point as 6.98. Furthermore, the secondary and tertiary structure of *Md*PGIP1 was modelled, and revealed that *Md*PGIP1 is a curved and elongated molecule that contains sheet B1, sheet B2 and 3_10_-helices on its LRR domain.

**Conclusion:**

The overall properties of the *Md*PGIP1 protein is similar to that of the prototypical *Phaseolus vulgaris* PGIP 2 (*Pv*PGIP2), and the detected differences supported its use in biotechnological applications as an inhibitor of targeted fungal polygalacturonases (PGs).

## Background

Polygalacturonase inhibiting proteins (PGIPs) are part of the innate immune system of plants. PGIPs may specifically inhibit fungal polygalacturonases (PGs) [1-3]. The action of PGIPs on PGs during fungal attack slows down the infection rate and facilitates the prolonged existence of mid-sized oligogalacturonides (damage-associated molecular pattern molecules, DAMPs), which in turn can elicit a general defence response from the plant [4-6]. PGIPs are not specialized inhibitors of a single PG, but rather versatile proteins that capable of recognising different surface motifs of structurally variable PGs [6]. Knowledge about the structural properties of PGIPs can provide valuable insight into the nature and dynamics of these interactions with fungal PGs, and also helps to identify promising candidate PGIPs for biotechnological approaches to improve plant disease resistance.

Yao *et al.* [7] isolated mRNA containing the complete coding sequence region of the *MdPGIP* gene from cDNA of *M. domestica* cv Golden Delicious [GenBank: MDU77041]. Similarly, Arendse *et al.* [8] isolated the complete *MdPGIP1* gene from gDNA of *M. domestica* cv Granny Smith [GenBank:DQ185063]. The sequence of the *MdPGIP1* accession DQ185063 was compared to the *MdPGIP* accession MDU77041 and the results showed that the two gene sequences share a 100% identity. The *MdPGIP1* gene sequence elicited interest in its potential use as an anti-fungal agent and was subsequently used to transfer into potato [9] and tobacco [10]. *Md*PGIP1 inhibits PGs from *Botryosphaeria obtuse* and *Diaporthe ambigua*, which are both apple pathogens. In addition, further studies performed to date (Matsaunyane and Oelofse, unpublished) indicate that the protein also inhibits PGs from *Verticillium dahlia, Botrytis cinerea*, *Colletotrichum acutatum* and *Colletotrichum coccodes*, but not that of *Fusarium verticillioides.* To further explore the biochemical characteristics of *Md*PGIP1 as a potential tool in improving disease resistance of food crops, *in silico* analyses were performed to compare the properties of *Md*PGIP1 to other characterised PGIPs. This further characterization forms part of new information on the *MdPGIP1* encoded protein.

## Methods

The *MdPGIP1* gene sequence with the GenBank accession [DQ185063] was used during *in silico* analysis in this study. The nucleotide sequence was translated into the encoding polypeptide using the http://web.expasy.org/translate/ database. The amino acid composition of the *Md*PGIP1 protein was calculated using the http://www.biology.arizona.edu/biochemistry/biochemistry.html database. In addition, the polarity, functional group side chains and their respective charge, and the amino acids’ water propensity, were also calculated on this database. The hydrophobicity plot of the *Md*PGIP1 contiguous amino acid residues was determined by constructing the Kyte-Doolittle hydropathy graph (http://gcat.davidson.edu/DGPB/kd/kyte-doolittle.htm) [11]. The molecular weight of *Md*PGIP1 was determined by compiling a ProtScale of the polypeptide using its respective constituting residues (http://web.expasy.org/cgi-bin/protscale/protscale.pl). Software from the NetNGlyc 1.0 Server (http://genome.cbs.dtu.dk/services/) was used to analyse the possible *N*-linked glycosylation sites of *Md*PGIP1. The putative crystal structure of *Md*PGIP1 was modelled using SWISS-MODEL [12], a protein structure homology-modeling server, accessible via the ExPASy web server (www. Swissmodel.expasy.org).

## Findings

### Amino acid composition and primary structure of *Md*PGIP1 supports its folded structure and function

**Figure 1:**
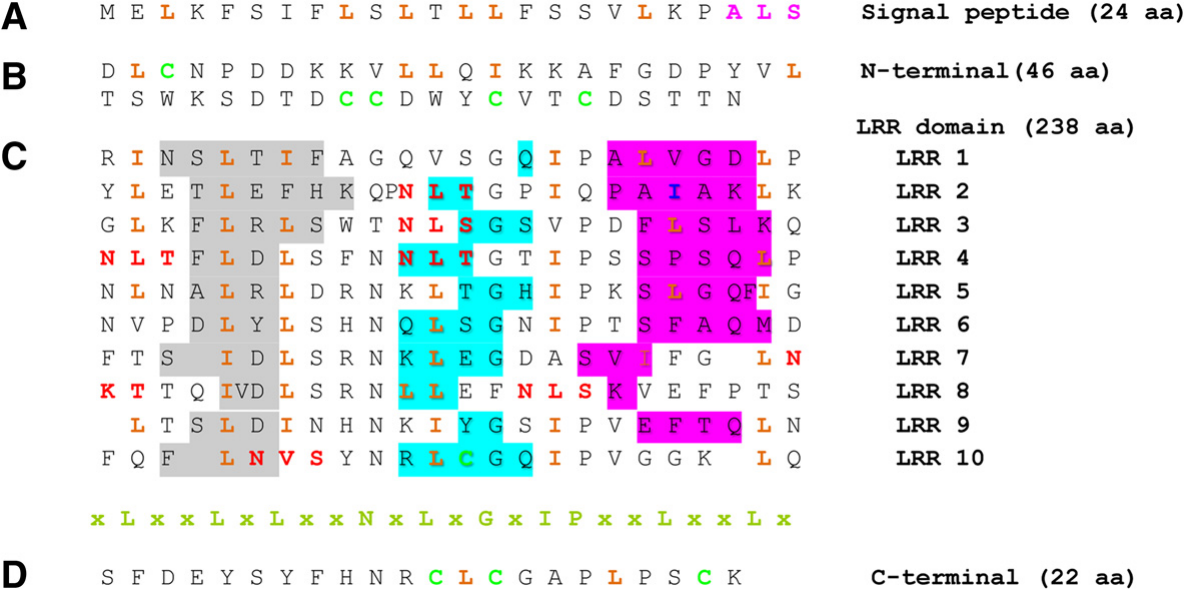
**The translated primary structure of the** ***Malus domestica*** **polygalacturonase inhibiting protein 1 (** ***Md*** **PGIP1).** Single letter codes are used to present amino acids. **A**: signal peptide, **B**: N-terminal domain, **C**: leucine-rich repeat LRR domain, and **D**: C-terminal domain. Hydrophobic amino acids leucine (L) and isoleucine (I) are highlighted in orange and the LRR consensus sequence is highlighted in lime. The N-glycosylation sites (N-X-S/T) are highlighted in red, with the cleavage site (A-L-S) highlighted in pink. N- and C-terminal cysteine residues are highlighted in sea green. Sheet-B1, sheet-B2 and 3_10_-helix are highlighted with grey, blue and pink background colours, respectively.

Although subject to modification, the linear sequence of amino acids, as represented by the primary structure of a protein, holds the required information for protein folding, the biological and cellular processes and activities of proteins. The amino acid residues that comprise the *Md*PGIP1 polypeptide were therefore analysed, based on the translated nucleotide sequence [13]. The primary structure of *Md*PGIP1 with its identified domains is shown in Figure 1. Features include a putative 24 amino acid signal peptide (Figure 1A), a potential cleavage site [Alanine-Leucine-Serine (ALS)] for the signal peptide (Figure 1, ALS in pink), a 46 amino acid N-terminal domain (Figure 1B), a 238 amino acid leucine-rich repeat (LRR) domain (Figure 1C) and a 22 amino acid C-terminal domain (Figure 1D).

**Figure 2:**
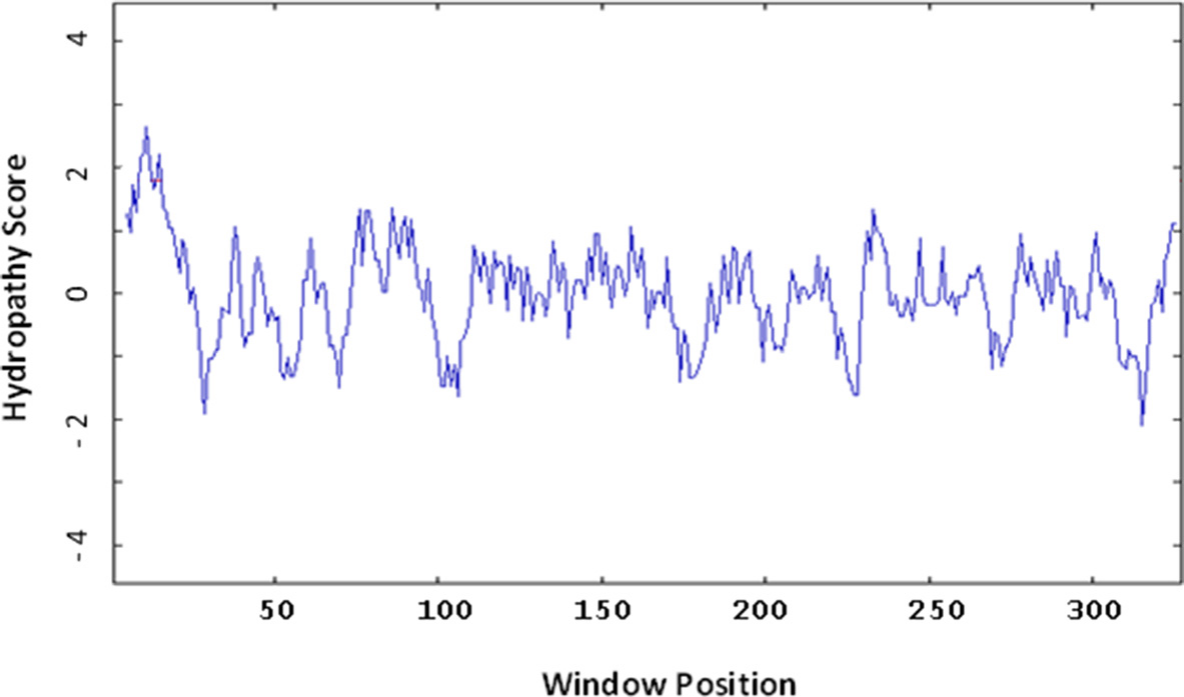
**A hydropathy plot constructed for the** ***Malus domestica*** **polygalacturonase inhibiting protein 1 (** ***Md*** **PGIP1) polypeptide.** The number of amino acids, of the total 330, that were effective in the construction of the hydropathy plot was 322 with a window size of 9.

The amino acid composition of the polypeptide, the polarity of the amino acids, type of side chain found in their respective functional (R) group, charge of the amino acids, as well as their water propensity was also calculated (http://www.biology.arizona.edu/biochemistry/biochemistry.html). The water propensity of amino acids can be used to indicate, *in silico*, the location of the respective amino acid in the final structure of a protein during folding [11]. The interior of a globular protein normally houses hydrophobic residues, whereas the outer side is a location for hydrophilic residues. The Kyte-Doolittle hydropathy graph was subsequently constructed (Figure 2) to obtain further insights into the effect of the different water propensities of the *Md*PGIP1 residues on the structure of the protein (http://gcat.davidson.edu/DGPB/kd/kyte-doolittle.htm, [11]). The total number of amino acids, of the total 330, that were effective in the construction of the hydropathy plot was 322. The window size of the *Md*PGIP1 hydropathy plot was 9 and strong negative peaks (indicative of hydrophilic areas) were observed on the plot. This is an indication of a possible surface area of a globular protein. The hydropathy plot also indicated the existence of a repetitive region between residues 71 and 300 on the window number (x-axis), and by the strong positive (hydrophobic) peaks on the hydrophobicity score (y-axis) (Figure 2).

PGIPs are known to be peripheral membrane-associated proteins secreted to the apoplast [14]. The hydropathy plot was used to identify portions of *Md*PGIP1 that could be associated with the membrane. The most hydrophobic residues served as a basis for this identification and these residues were found from residue 9 to 21 on the protein [11]. The residues found at this location on *Md*PGIP1 are Met, Glu, Leu, Lys, Phe, Ser, Ile, Phe, Leu, Ser, Leu, Thr, Leu, Leu, Phe, Ser, Ser, Val, Leu, Lys, Pro, Ala, Leu and Ser (Figure 1). The relative hydrophobicity of this portion is confirmed in Figure 2, where the hydropathy score is the highest throughout the span of the *Md*PGIP1 polypeptide length at a value between one and two. This hydropathy score is thought to be a deciding factor for this portion to be membrane associated, thus confirming this aspect for *Md*PGIP1, similar to other PGIPs [15].

### *Md*PGIP1 belongs to the Leucine Rich Repeat superfamily

The structural features of *Md*PGIP1 were found to be consistent with typical PGIP features described by other authors [16-19]. Structural studies of PGIP proteins are important for it is known that the change of one or a few residues may confer new PGs recognition specificities to a PGIP and may improve its inhibitory strength [6,20]. The recognition specificity is determined by variation in the amino acids comprising the LRR domain [6,20]. This data may support the planning of mutational strategies towards improving the properties of natural PGIPs and the versatility of their recognition capabilities against the many diverse microbial PGs [21].

As mentioned, evaluation of the primary structure of *Md*PGIP1 indicates a LRR region of 238 amino acids containing 123 hydrophobic amino acids (Figure 1). The LRR domain spans from residues 71 to 308. The *Md*PGIP1 polypeptide contains 10 LRRs as indicated in Figure 1, hence the protein belongs to the LRR family [16,18,22]. All the PGIPs isolated to date comprise 10 LRRs matching the extracytoplasmic LRR consensus LxxLxxLxxLxLxxNxLxGxIPx, features that also apply to *Md*PGIP1.

LRR motifs play an important role in the cellular functions of several proteins [23]. A typical motif contains 20 to 29 residues and these motifs have been identified in plants, animals, as well as in microorganisms [20,23]. In the case of PGIP, these leucine residues are important in the binding of PGIP to the cell wall through the interaction of the residues with the acidic pectin within the cell wall matrix [14,15].

### Physicochemical properties and glycosylation of the *Md*PGIP1 pre-protein

Following the analysis of how hydrophobicity and hydrophilicity affect the *Md*PGIP1 structure, further analyses were performed to determine the molecular weight of *Md*PGIP1. ProtScale was used to compute the profile of *Md*PGIP1 and produced its constituent amino acids (http://web.expasy.org/cgi-bin/protscale/protscale.pl). The molecular weight of all the residues that make up *Md*PGIP1 was determined per residue and mapped on the polypeptide to assist with determining the molecular weight of the protein (graph not shown).

Purified *Md*PGIP1 was found to have a molecular mass of between 44 to 54 kDa (cv Golden Delicious) [24] and 46 kDa (cv Granny Smith) [10]. Using the residues’ molecular mass and their mapping throughout the *Md*PGIP1 polypeptides, the molecular mass of the non-glycosylated apo-protein was calculated and determined to be 36.615 kDa. The bulk of the *Md*PGIP1 polypeptide consists of Leu and Ser at 16.1% and 10.6%, respectively (Figure 1), a combined contribution of 29% to the molecular weight of *Md*PGIP1.

Lastly, the theoretical isoelectric point value (pI) of *Md*PGIP1 was calculated to be 6.98. The relatively high pI of the mature *Md*PGIP1 polypeptide is attributed to the presence of the positively charged Lys and Arg residues. These residues are believed to interact with the acidic pectin in the cell wall matrix, supporting the cell wall association of PGIP [16]. Addition of amino sugars during glycosylation of the pre-protein can further increase the pI to 8.0 [10], generating a basic functional protein.

PGIPs have been reported to be glycoproteins [4,22,25], undergoing post-translational glycosylation [26,27]. Glycosylation enables proteins to participate in biological processes, such as attaching to the extracellular matrix, as well as protein-ligand interactions, and has been shown to contribute to protein stability and increase resistance to protease digestion [27]. These are important features related to the function of PGIP in an extracellular environment.

*N*-glycosylation initially occurs in the endoplasmic reticulum and the target residue is Asp that is found in the sequence Asn-X-Ser/ Thr, where X can be any residue except Pro [28,29]. The *N*-linked glycosylation sites of *Md*PGIP1 were analysed using the NetNGlyc 1.0 Server (http://genome.cbs.dtu.dk/services/) and the analysis is shown in Table 1. The typical consensus glycosylation sequence required for this modification (Asn-X-Ser/Thr) was identified in *Md*PGIP1, supporting its characterization as a glycoprotein. Aspects of the glycosylation process that can be modified and thus affect biological function, include glycan composition, glycan structure and glycan length [30].

**Table 1:** **The calculated N-linked glycosylation sites found on the** ***Malus domestica*** **polygalacturonase inhibiting protein 1 (** ***Md*** **PGIP1) polypeptide**

**Position**	**Possibility**	**N-Glycosylation**
106 NLTG	9/9	++
130 NLSG	8/9	+
144 NLTF	5/9	-
154 NLTG	8/9	+
238 NKTT	8/9	+
254 NLSK	9/9	++
291 NVSY	9/9	++

The position of the receptor sites for *N*-glycosylation is included. The possibility of glycosylation occurring at that position is indicated with a + if it is positive, and - for highly unlikely.

### Sequence comparison of the *Md*PGIP1 encoded protein

**Figure 3:**
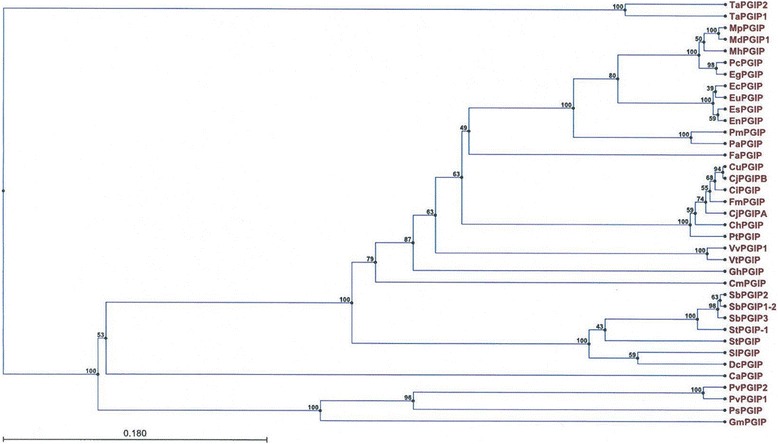
**A phylogenetic tree representing the hierarchical clustering of the pairwise similarities between polygalacturonase inhibiting proteins (PGIPs)**. The tree was constructed using the unweighted pair group method with arithmetic mean (UPGMA) and bootstrapping was also performed to verify the accuracy of the pairwise similarities identified. *Triticum aestivum2* (*Ta*PGIP2; AM180657), *T. aestivum*1 (*Ta*PGIP1; AM180656), *Malus pumila* (*Mp*PGIP; JQ001783), *M. domestica* PGIP (*Md*PGIP; U77041), *M. hupehensis* (*Mh*PGIP; FJ449708), *Pyrus communis* PGIP (*Pc*PGIP; L09264), *Eucalyptus grandis* (*Eg*PGIP, AY445043), *E. camaldulensis* (*Ec*PGIP; AF159168), *E. urophylla* (*Eu*PGIP; AF159169), *E. saligna* (*Es*PGIP; AF159170), *E. nitens* (*En*PGIP; AF159171), *Prunus mahaleb* (*Pm*PGIP; AF263465), *P. americana* (*Pa*PGIP; AY883418), *Fragaria ananassa* (*Fa*PGIP; EU117215), *Citrus unshiu* (*Cu*PGIP; AB016204), *C. jambhiri*B (*Cj*PGIPB, AB015198), *C. iyo* (*Ci*PGIP, AB016205), *Fortunella margarita* (*Fm*PGIP; AB020529), *C. jambhiri*A (*Cj*PGIPA, AB013397), *C. hystrix* (*Ch*PGIP; AB071018), *Poncirus trifoliata* (*Pt*PGIP, AB050528), *Vitis vinifera*1 (*Vv*PGIP1; AF499451), *V. thunbergii* (*Vt*PGIP; EU037367), *Gossypium hirsutum* (*Gh*PGIP; EU711352), *Cucumis melo* (*Cm*PGIP; KC215471), *Solanum brevidens*2 (*Sb*PGIP2; DQ185391), *S. brevidens*1-2 (*Sb*PGIP1-2; DQ185393), *S. brevidens*3 (*Sb*PGIP3; DQ185392), *S. tuberosum*1 (*St*PGIP1; AY662681), *S. torvum* (*St*PGIP, FJ943498), *S. lycopersicum* (*Sl*PGIP; L26529), *Daucus carota* (*Dc*PGIP; AF055480*), Capsicum annuum* (*Ca*PGIP; EU560898), *Phaseolus vulgaris*2 (*Pv*PGIP2, AJ864507), *P. vulgaris*1 (*Pv*PGIP1; X64769), *Pisum sativum* (*Ps*PGIP; AJ749705) and *Glycine max* (*Gm*PGIP; X78274).

PGIPs have been identified in many dicot and monocot plants. *PGIP* genes are often found as small gene families that encode PGIP isoforms with different specificities and affinities towards secreted fungal PGs [6]. The phylogenetic relationship between *Md*PGIP1 was compared with PGIPs from other plants as shown in Figure 3. Amino acid comparisons between the different PGIPs showed that PGIPs from fruit bearing trees share a high percentage of similarity with each other when compared to other plants. *Md*PGIP1 shares a 99.4%, 98%, and 98% amino acid identity with the PGIPs from *Malus pumila*, *Malus hupehensis* and *Pyrus communis* (members of the Rosaceae family), respectively.

The *Md*PGIP1 amino acid sequence was also compared PGIPs from non-fruit bearing *Eucalyptus* tree species (members of the Myrtaceae family), namely, *E. grandis*, *E. saligna*, *E. nitens*, *E. urophylla* and *E. camaldulensis*. Interestingly, *Md*PGIP1 was found to share a 97% amino acid identify with *E. grandis*, and a 96% amino acid identity with *E. saligna*, and *E. nitens. E. urophylla* and *E. camaldulensis* share a 95% amino acid identity with *Md*PGIP1. In contrast, *Md*PGIP1 shares only a 55%, 48% and 53% identity with *Pv*PGIP1, *Pv*PGIP2 and *Gm*PGIP (members of the Fabaceae family) respectively.

An analysis of PGIP sequences from different eudicotyledonous species (Fabaceae, Brassicaceae, Rosaceae and Rutaceae) indicated that diversification of *PGIP* genes during evolution has been driven by positive selection [31], limited to a small number of PGIP residues that are mostly solvent exposed and located in the β-sheet B1 corresponding to the concave surface of the protein (below).

### Structural modeling: *Md*PGIP1 shares a similar structure with *Pv*PGIP2

Two types of repeats, types A and B, are found in plant LRR domains [23]. Eight repeats of 28 amino acids were found in type A and 29 amino acids in type B. Short β-strand, βα loop, α helix and βα loop regions are formed by the type A repeats. This leads into repeats that form a parallel β-strand. The type A formation is repeated until the C-terminal, ending with a β-strand. β-Sheets are formed parallel to the α helix on the face of the protein. These sheets are formed by stabilising residues found on adjacent repeats. The formation of β-sheets creates curvature to the protein and gives it a horseshoe shape.

**Figure 4:**
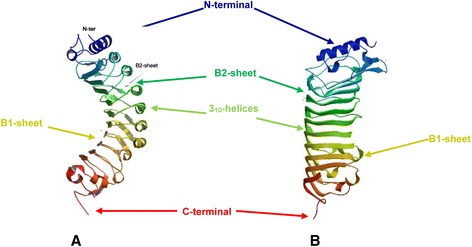
**The ribbon representation of the folded structure of the** ***Malus domestica*** **polygalacturonase inhibiting protein 1 (** ***Md*** **PGIP1) in comparison to** ***Phaseolus vulgaris*** **PGIP2 (** ***Pv*** **PGIP2). A**: *Pv*PGIP2 structure, and **B**: *Md*PGIP1 structure. PGIPs have evolved a wide interacting surface within the concave face of the LRR domain that is subject to evolutionary pressure for diversification [2,31,33].

The molecular structure of PGIP2 from *Phaseolus vulgaris* (*Pv*PGIP2) was elucidated using X-ray crystallography (Figure 4A) [20,32]. Although the presence of a single β-sheet in *Pv*PGIP2 was predicted, it was shown that two β-sheets (sheet B1 and B2) were present in *Pv*PGIP2. In addition to the two β-sheets, helices were also found on the LRR domain of the *Pv*PGIP2 molecule. The protein structure was found to be curved and elongated which is typical of other PGIPs [23]. The residues found in the β-strand/β-turn motif of PGIP were reported to be critical in the protein’s affinity and specificity towards PG ligands [20,32].

The crystal structure of *Pv*PGIP2 served as a template to which the *Md*PGIP1 was modelled. The putative structure of *Md*PGIP1 was modelled using SWISS-MODEL [12] and the modeling results are shown in Figure 4B. The structure of *Md*PGIP1 was also found to be curved and elongated. In addition, sheet B1, sheet B2 and 3_10_-helices were also identified on the LRR domain of the *Md*PGIP1 molecule.

**Figure 5:**
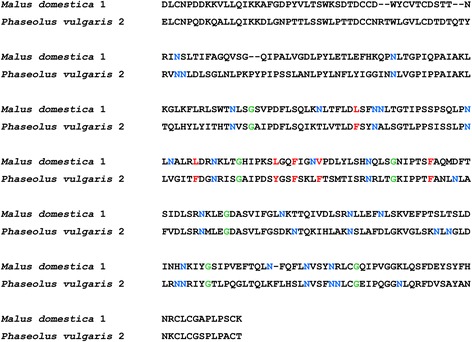
**Alignment of the amino acids from** ***Phaseolus vulgaris*** **polygalacturonase inhibiting protein 2 (** ***Pv*** **PGIP2) and** ***Malus domestica*** **PGIP1 (** ***Md*** **PGIP1) without the signal peptides.** Glycine residues and aromatic and hydrophobic residues thought to be responsible for the bending of sheet B2 in *Pv*PGIP2 are highlighted in green and red, respectively. Asparagine residues are highlighted in blue within the LRR domains.

Sheet B1 of the *Md*PGIP1 LRR domain is located near the N-terminal in the concave inner side of the LRR region (Figure 4). The *Md*PGIP1 sheet B1 comprises 38 residues, of which 19 are hydrophobic, and these are located at residues 75, 77–78, 99, 101, 123–124, 126, 147–148, 171–172, 197, 220, 243–244, 267, 289 and 290 on the *Md*PGIP1 polypeptide (Figure 5). The hydropathy plot confirmed the observation where the hydrophobicity scores at these residue positions are relatively high. Sheet B2 is located on the convex outer side of the LRR region and comprises 29 residues, with 16 of those being hydrophilic (Figure 5). These hydrophilic residues are found at position 85, 108, 132, 134, 154, 156, 180, 182, 203, 205, 226, 228, 275, 296, 298 and 300 on the *Md*PGIP1 polypeptide. This water propensity of the sheet B2 residues is confirmed on the hydropathy plot. Sheet B2 determines the folding of PGIPs by connecting β-sheet B1 and 3_10_-helices [32]. In addition, it is thought to form an additional surface on the PGIP for interaction with PGs [33].

Glycine residues found in sheet B2 of PGIPs are thought to be responsible for the bending of the sheet [32,33] and these residues were identified in the analysis of *Md*PGIP1 (Figure 5, green highlighted residues). Further analysis of the glycine residues revealed that they are located, in sheet B2 of *Md*PGIP1 (Figure 5, green highlighted residues), at positions similar to those identified on *Pv*PGIP2. The speculation is that the *Md*PGIP1 sheet B2 glycine residues are also responsible for the bending of the sheet, as was described for *Pv*PGIP2 [20,32]. However, the *Md*PGIP1 molecule does have a curve that is more relaxed compared to *Pv*PGIP2.

The amino acids within sheet B2 of *Pv*PGIP2 also comprise several aromatic and hydrophobic residues that contribute to the bending of the *Pv*PGIP2 molecule [32,33]. These residues were identified as Phe-133, Phe-156, Phe-172, Phe-176, Phe-194, and Tyr-169. During analysis of the *Md*PGIP1 polypeptide, amino acids identified at the same positions were Leu-133, Leu-156, Phe-172, Val-176, Phe-194, and Leu-169. Leucine and valine are hydrophobic aliphatic residues with smaller side chains and these residues may be the cause of the more relaxed curve observed on the *Md*PGIP1 molecule.

While sheet B2 is thought to form an additional surface on the PGIP protein for interaction with PGs [32,33], sheet B1 residues determine the affinity and specificity of *Pv*PGIP2. Asparagine residues have been found to form an Asn-ladder on *Pv*PGIP2 and these were found to form hydrogen bonds with amide groups and the main-chain carbonyl. This quality also influences the bending of the protein [32,33]. Twelve of the 20 Asn residues observed on the *Pv*PGIP2 molecule were also observed on *Md*PGIP1 (Figure 5, blue highlighted residues).

## Conclusion

Although phylogenetically distant from the archetypal *Pv*PGIP2, the overall properties of the *Md*PGIP1 protein are broadly similar to that of the PGIPs thus far characterised. However, even slight structural differences may confer new or broader recognition specificities to a PGIP or may improve its inhibitory strength. Based on the foundation laid in the present study, future studies of the detected differences will add support to the biotechnological use of *Md*PGIP1 in recombinant transgenic applications as a targeted inhibitor of fungal PGs. Moreover, it may assist in the identification of promising candidate PGIPs for crop protection, and in improving the properties of natural PGIPs and thus the versatility of their recognition capabilities against the many diverse microbial PGs.

## Abbreviations

A-L-S: Signal peptide cleavage site
DAMP: Damage-associated molecular pattern
LRR: Leucine-rich repeat
N-X-S/T: N glycosylation site
PG: Polygalacturonase
PGIP: Polygalacturonase inhibiting protein
UPGMA: Unweighted pair group method with arithmetic mean
